# CD106 is a novel mediator of bone marrow mesenchymal stem cells via NF-κB in the bone marrow failure of acquired aplastic anemia

**DOI:** 10.1186/s13287-017-0620-4

**Published:** 2017-08-01

**Authors:** Shihong Lu, Meili Ge, Yizhou Zheng, Jianping Li, Xiaoming Feng, Sizhou Feng, Jinbo Huang, Ying Feng, Donglin Yang, Jun Shi, Fang Chen, Zhongchao Han

**Affiliations:** 1 State Key Laboratory of Experimental Hematology, Institute of Hematology and Blood Diseases Hospital, Chinese Academy of Medical Science and Peking Union Medical College, 288 Nanjing Road, Tianjin, 300020 People’s Republic of China; 2 Department of Hematology, Qinghai Provincial People’s Hospital, Xining, Qinghai China

**Keywords:** Aplastic anemia, Mesenchymal stem cells, CD106, hematopoiesis

## Abstract

**Background:**

Acquired aplastic anemia (AA) is characterized by deficiency or dysfunction of the bone marrow (BM) microenvironment. However, little is known about the impairment of BM-derived mesenchymal stem cells (MSCs) in AA patients.

**Methods:**

We used Illumina HiSeqTM 2000 sequencing, quantitative real-time polymerase chain reaction (qRT-PCR), flow cytometry (FCM), and Western blotting to test the expression of CD106 gene (vascular cell adhesion molecule 1 (VCAM1)) and CD106 protein of BM-MSCs. Furthermore, we used hematoxylin and eosin (H&E) and histochemical staining analysis, immunofluorescence, and the formation of capillary-like structures to analyze capillary tube-like formation in vitro; we also used the Matrigel plug assay to test in vivo vasculogenesis, and an assay of colony forming units (CFUs) and colony-forming unit-megakaryocyte (CFU-MK) to detect the support function of MSCs in vitro. The in vivo engraftment of CD34^+^ cells and MSCs in NOD/SCID mice was tested by FACS and survival assay; the expression of NF-κB was tested by NanoPro analysis and immunofluorescence. NF-κB-regulated CD106 gene (VCAM1) was confirmed by tumor necrosis factor alpha (TNF-α)-stimulated and lipopolysaccharide (LPS)-stimulated MSCs, blockade assay, and immunofluorescence.

**Results:**

Here, we report that BM-MSCs from AA patients exhibited downregulation of the CD06 gene (VCAM1) and low expression of CD106 in vitro. Further analysis revealed that CD106^+^ MSCs from both AA patients and healthy controls had increased potential for in vitro capillary tube-like formation and in vivo vasculogenesis compared with CD106^–^ MSCs, and the results were similar when healthy MSCs were compared with AA MSCs. CD106^+^ MSCs from both AA patients and healthy controls more strongly supported in vitro growth and in vivo engraftment of CD34^+^ cells in NOD/SCID mice than CD106^–^ MSCs, and similar results were obtained when healthy MSCs and AA MSCs were compared. The expression of NF-κB was decreased in AA MSCs, and NF-κB regulated the CD106 gene (VCAM1) which supported hematopoiesis.

**Conclusions:**

These results revealed the effect of CD106 and NF-κB in BM failure of AA.

**Electronic supplementary material:**

The online version of this article (doi:10.1186/s13287-017-0620-4) contains supplementary material, which is available to authorized users.

## Background

Aplastic anemia (AA) is generally considered an immune-mediated bone marrow (BM) failure syndrome characterized by hypoplasia, pancytopenia with fatty BM, and reduced angiogenesis [1–3]. Acquired AA is also associated with abnormalities in hematopoietic stem/progenitor cells (HSCs/HPCs) and the hematopoietic microenvironment, which are mediated by abnormal immunity [2].

Mesenchymal stem cells (MSCs) residing in the BM are critical for HSC niche formation in the BM microenvironment. BM-MSCs can differentiate into a variety of cells, including endothelial cells, adipocytes, fibroblasts, and osteoblasts, which constitute the HSC niche, support hematopoiesis, and regulate the function of almost all immune cells to maintain hematopoiesis and immune homeostasis [4].

Studies have shown that MSCs are deficient in terms of proliferation, differentiation, and hematopoietic support in acquired AA [1–3]. However, the underlying molecular mechanism is not yet well defined. Defective angiogenesis, such as decreased expression of angiopoietin-1 (ANG-1) and vascular cell adhesion molecule-1 (VCAM1 or CD106) genes, has also been demonstrated in acquired AA, indicating abnormal regulatory patterns in the osteoblastic and vascular niches [5]. However, it remains unclear whether defective angiogenesis in AA is associated with abnormal function or differentiation of MSCs.

CD106 (VCAM1) is a cytokine-inducible cell surface protein capable of mediating adhesion. A previous study showed that CD106-deficient (CD106^–^) mouse embryos were not viable and exhibited one of two distinct phenotypes. Half of the embryos died before embryonic day 11.5 and exhibited severe defects in placental development, and the remaining embryos survived until embryonic day 11.5–12.5 and displayed several abnormalities in heart development [6]. CD106 is a component of the neural stem cell niche [7] that is critical for MSC-mediated immunosuppression [8, 9] and HPC binding [10]. However, little is known about the quantity and function of CD106 in BM-MSCs from AA patients.

NF-κB is composed of multiple distinct subunits. In vivo, NF-κB is activated by a variety of stimulants, such as tumor necrosis factor (TNF)-α, interleukin (IL)-1, and lipopolysaccharide (LPS). The role of the specific subunits in CD106 gene expression has been defined. Biochemical and molecular analyses have indicated that NF-κB binds to the κB sites as a heterodimer or a homodimer. At least five cDNAs encoding NF-κB subunits—nfkbl, nfkb2, rel4, c-rel, and relB—have been isolated. In most cases, NF-κB binds as a 50-kDa heterodimer generated from either NF-κB1(p105) or NF-κB2(p100) in combination with RelA(p65) to stimulate gene expression. The CD106 gene enhancer responds to combinations of NF-κB subunits that are distinct from other promoters, demonstrating that specific combinations of NF-κB can selectively regulate CD106 (VCAM1) gene expression in vivo [11].

The present study was designed to investigate the role of CD106^+^ MSCs in the pathogenesis of acquired AA. We found abnormal expression of a large number of genes, including CD106 (VCAM1), C-X-C motif chemokine 12 (CXCL12), chemokine ligand 2 (CCL2), and IL-6 genes in BM-MSCs of AA patients. Furthermore, we observed a significant reduction in CD106^+^ MSCs from AA patients, and CD106^–^ MSCs from both AA patients and healthy controls were less potent than CD106^+^ MSCs in terms of differentiation, hematopoietic support, and angiogenesis in vivo and in vitro. The profile and quantitation of NF-κB was decreased in BM-MSCs from AA patients compared with those in BM-MSCs from healthy controls. When NF-κB was blocked, CD106 protein expression was downregulated. Our study implicates CD106 and NF-κB in the pathogenesis of AA.

## Methods

### Patients

BM samples from 28 (17 male and 11 female) de novo acquired AA patients with a median age of 31 years (range 18–59 years) were analyzed after the signing of a written informed consent form in accordance with the Declaration of Helsinki. The study was approved by the Committee for Medical Care and Safety, Institute of Hematology and Blood Diseases Hospital, Chinese Academy of Medical Science and Peking Union Medical College (ethical approval documents reference number KT2014005-EC-1). This cohort consisted of four patients with severe AA and 24 with nonsevere AA. The diagnosis and severity classification of AA was established by morphological examination of the BM and peripheral blood samples after excluding any other acquired BM failure syndromes, such as paroxysmal nocturnal hemoglobinuria, myelodysplastic syndrome, and congenital BM failure syndromes, according to international criteria [12]. Samples from 19 (14 male and 5 female) age-matched (range 20–56 years) healthy controls were obtained after they had signed the written informed consent form described above.

### Animals

Our experimental research on NOD/SCID and nude mice followed internationally recognized guidelines. Ethical approval for the animal experiments was provided by the Ethical Committee of the Institute of Hematology and Blood Diseases Hospital, Chinese Academy of Medical Science and Peking Union Medical College. The ethical approval documents reference number is KT2012003-m-6.

### Isolation and identification of BM-MSCs

BM-MSCs were isolated and cultured in Dulbecco’s modified Eagle’s medium [13]. BM-MSCs were identified by their surface markers with a panel of monoclonal antibodies against CD13 (WM15), CD29 (MAR4), CD44 (G44-26), CD49e (IIA1), CD73 (AD2), CD105 (266), CD166 (3A6), CD31 (WM59), CD34 (581), CD45 (HI30), CD90 (5E10), HLA-ABC (G46-2.6), HLA-DR (G46-6), CD14 (M5E2), CD40 (5C3), and CD11b (ICRF44), along with the appropriate isotype monoclonal antibodies using a FACScanflow cytometer (BD Biosciences, San Jose, CA, USA).

### Illumina HiSeqTM 2000 sequencing

RNA samples were first treated with DNase I to degrade any possible DNA contamination. Next, mRNA was enriched using oligo (dT) magnetic beads for eukaryotes and fragmented into short fragments of approximately 200 bp. The first strand of cDNA was synthesized using a random hexamer-primer, and then buffer, dNTPs, RNase H, and DNA polymerase I were added to synthesize the second strand. Double-stranded cDNA was purified with magnetic beads, followed by end reparation and 3’-end single nucleotide A (adenine) addition. Finally, sequencing adaptors were ligated to the fragments, which were enriched using polymerase chain reaction (PCR) amplification. A sample library was qualified and quantified using an Agilent 2100 Bioanalyzer (Agilent, Santa Clara, CA, USA) and an ABI StepOnePlus Real-Time PCR System (Applied Biosystems, Carlsbad, CA, USA) during the quantitative-competitive (QC) step. Library products were ready for sequencing via Illumina HiSeqTM 2000 (Illumina, San Diego, CA, USA) or other sequencer when necessary. Next, quantitative real-time polymerase chain reaction (qRT-PCR) was performed to confirm the gene expression levels of RNA transcripts with sequence-specific oligonucleotide primers as described previously.

### Separation of CD106^+^ MSCs and CD106^–^ MSCs

MSCs were labeled with PE-conjugated anti-CD106 antibody (BD Biosciences). CD106^+^ MSCs and CD106^–^ MSCs were separated using a CD106-positive selection magnetic-activated cell sorting (MACS) isolation kit (Miltenyi Biotech, Bergisch Gladbach, Germany) according to the manufacturer’s instructions. CD106^+^ MSCs or CD106^–^ MSCs (≥90% purity) were used for subsequent experiments.

### Flow cytometry (FCM)

Cells were stained with antibodies along with the appropriate isotype controls (BD Biosciences) according to the manufacturer’s instructions. Data acquisition was performed using an LSR II flow cytometer (BD Biosciences) and analyzed with FlowJo 7.6 software (FlowJo, Ashland, OR, USA).

### Hematoxylin and eosin (H&E) and histochemical staining analysis

H&E staining (Sigma-Aldrich) and histochemical staining (Abcam, Cambridge, UK) were performed according to the manufacturers’ instructions. Samples were photographed using a Nikon ElipseTi-U microscope (Nikon, Tokyo, Japan).

### Immunofluorescence

The expression of cell surface molecules was assessed according to the manufacturer’s instructions. Normal MSCs (N MSCs) were stained for CD106 and NF-kB. N MSCs were first washed and fixed with 4% formaldehyde for 15 min and then blocked with blocking buffer (phosphate-buffered saline (PBS)/5% normal serum) for 60 min. N MSCs were labeled with mouse-anti-human CD106 overnight and then labeled with Alexa Fluor® 546-goat anti-mouse IgG conjugated secondary antibody for 60 min. N MSCs with 0.3% Triton™ X-100 for 60 min were labeled with rabbit-anti-human NF-κB overnight and then labeled with Alexa Fluor® 488-conjugated secondary antibody (donkey anti-rabbit) for 60 min. The nucleus was marked with DAPI.

Selected CD106^+^/CD106^–^ MSCs were stained for NF-κB and nuclei to examine the NF-κB expression level. The expression of NF-κB was calculated as the mean of the fluorescence intensity in 6 continuous views. CD106^+^/CD106^–^ MSCs were first washed and fixed with 4% formaldehyde for 15 min and then blocked with blocking buffer (PBS/5% normal serum/0.3% Triton™ X-100) for 60 min. Then, CD106^+^/CD106^–^ MSCs were labeled with rabbit-anti-human NF-κB overnight and then labeled with Alexa Fluor® 488-conjugated secondary antibody or Alexa Fluor® 546-conjugated secondary antibody for 60 min. The nucleus was marked with DAPI.

Samples were photographed using an UltraVIEWVoX Confocal Imaging System (PerkinElmer, Waltham, MA, USA).

### Western blotting

Western blotting procedures were performed according to the protocol described by Song et al. [14]. Briefly, BM-MSCs were collected, washed, and lysed with RIPA lysis buffer (Beyotime Institute of Biotechnology, Shanghai, China) supplemented with PMSF (Invitrogen, Carlsbad, CA, USA). Total protein was extracted and quantified by the BCA protein assay kit (Pierce, Woodland Hills, CA, USA). A total of 30 μg protein was denatured, separated by SDS-PAGE electrophoresis, and transferred to a PVDF membrane. The transferred membranes were blocked using 5% bovine serum albumin (BSA) in TBST, incubated with anti-human CD106 mouse monoclonal antibody (Abcam) overnight, and then incubated with the corresponding horseradish peroxidase (HRP)-conjugated secondary antibody at a dilution of 1:2000 for 2 h. Bands were visualized using enhanced chemiluminescence (ECL; Thermo-Fisher, Scientific, Waltham, MA, USA) detection reagents, and scanned images were quantified using Image J (https://imagej.nih.gov/ij/). The ratio of target gene to β-actin was used for the semiquantification and comparison between the two groups.

### Formation of capillary-like structures

Wells in 96-well plates were covered with 50 μl of growth factor-reduced Matrigel (BD Biosciences). Aliquots of CD106^+^ MSCs and CD106^–^ MSCs were seeded at a density of 10,000 cells/cm^2^ and cultured in a humidified atmosphere with 5% CO_2_ for 24 h. The formation of capillary-like structures was observed using an Olympus IX71 inverted microscope (Olympus, Tokyo, Japan), and pictures were taken at different time points using an Olympus DP71 camera (Olympus).

### Matrigel plug assay

To confirm our in vitro data, we examined vasculogenesis in vivo by performing a Matrigel plug assay. Aliquots of 5 × 10^5^ MSCs were resuspended in 500 μl of Matrigel (BD Biosciences) according to the manufacturer’s instructions and implanted into the back of 42-day-old nude mice (*n* = 6 in each group). Mice implanted with Matrigel only were used as negative controls. After 21 days, the Matrigel plugs were harvested, assayed for microvessels identified as luminal structures with red blood cells using H&E staining, and counted.

### Detection of cytokine levels

Supernatants obtained from MSC-conditioned medium were used to detect vascular endothelial growth factor (VEGF) levels using an enzyme-linked immunosorbent assay (ELISA; R&D Systems, Minneapolis, MN, USA) according to the manufacturer’s instructions.

### Purification of CD34^+^ cells

CD34^+^ cells were freshly purified from umbilical cord blood (UCB) using a CD34/MACS isolation kit (MiltenyiBiotec, Bergisch, Gladbach, Germany) according to the manufacturer’s instructions. Cell fractions with 95 ± 5% CD34^+^ cell purity was used for subsequent experiments.

### Co-culture of CD34^+^ cells with MSCs

A total of 5 × 10^4^ CD34^+^ cells suspended in 1 ml of serum-free StemSpan™ H3000 (Stem Cell Technologies, Vancouver, Canada) culture medium was applied to feeder layers composed of 5 × 10^4^ BM-MSCs, as described previously. Cocultures were incubated for 14 days, and the culture medium was replenished every 3.5 days. Nonadherent viable cells were stained for FCM analysis using the antibodies anti-CD34-APC, anti-CD61-PE, anti-CD41a-PE, and anti-CD42b-PE, along with the appropriate isotype controls (BD Biosciences) according to the manufacturer’s instructions. Data acquisition was performed using an LSR II flow cytometer (BD Biosciences) and analyzed with FlowJo7.6 software (FlowJo).

A total of 1 × 10^5^ CD34^+^ cells suspended in serum-free StemSpan™ H3000 (Stem Cell Technologies) culture medium was applied to feeder layers composed of BM-MSCs, which were blocked by BAY-11-7082, as described previously. Cocultures were incubated for 10 days, and the culture medium was replenished every 3.5 days. Nonadherent viable cells were stained for FCM analysis using CD34-APC along with the appropriate isotype controls (BD Biosciences) according to the manufacturer’s instructions. Data acquisition was also performed using an LSR II flow cytometer (BD Biosciences) and analyzed with FlowJo7.6 software (FlowJo).

### Assay of colony forming units (CFUs)

Cocultures of CD34^+^ cells with MSCs or blocked MSCs were incubated for 14 days or 10 days. Nonadherent viable cells (2.5 × 10^2^ or 5 × 10^2^ in each well of a 24-well-plate) were plated on 0.5 ml of methylcellulose medium (Stem Cell Technologies) to evaluate the in vitro effects of MSCs on CFU growth. Colonies of >50 cells were scored after 14 days of culture. Experiments were performed in triplicate.

### CFU-megakaryocyte (CFU-MK) assay

Cocultures of CD34^+^ cells with MSCs were incubated for 14 days. Nonadherent viable cells (5 × 10^2^ in each well of a 24-well-plate) were plated on semisolid Iscove’s modified Dulbecco’s medium (IMDM; Gibco) supplemented with 1% methylcellulose, 10% fetal bovine serum (FBS), 1% BSA, 10^–4^ M mercaptoethanol, 2 mM l-glutamine, and 100 ng/ml thrombopoietin. Cultures were incubated at 37 °C in a humidified atmosphere with 5% CO_2_. CFU-MK colonies were identified after 14 days of culture under an Olympus IX71 inverted microscope (Olympus), and typical colonies were selected for immunostaining. Cell smears were prepared using Cytospin (Thermo-Fisher Scientific), stained with anti-CD41a antibody, and observed using an UltraVIEWVoX Confocal Imaging System (PerkinElmer).

### Co-transplantation of CD34^+^ cells and MSCs in NOD/SCID mice

Aliquots of cell preparations containing 2 × 10^5^ CD106^+^ MSCs or CD106^–^ MSCs and 1 × 10^5^ UCB CD34^+^ cells in 15 μl of Roswell Park Memorial Institute (RPMI) 1640 medium were injected into the tail vein of 28- to 35-day-old NOD/SCID irradiated mice (*n* = 6 for each pair of cells) using a Hamilton syringe. Some low-dose irradiated mice (320 cGy) were sacrificed 42 days after xenotransplantation. CD45^+^ cells were analyzed after staining with human anti-CD45-APC antibody along with the appropriate isotype controls (BD Biosciences) according to the manufacturer’s instructions. Some high-dose irradiated mice (360 cGy) (*n* = 6 for each pair of cells) were observed until 56 days after xenotransplantation or death.

### Blockade assay

BM-MSCs were blocked by incubation with 500 ng/ml of the CD106 blocking antibody ab47159 for 1 h at 4 °C. BM-MSCs were blocked by incubation with 10 nM of the NF-κB-specific inhibitor BAY-11-7082 for 0.5 h, 1 h, or 12 h.

### NanoPro analysis for NF-κB

Cells were lysed in Bicine/CHAPS Lysis Buffer (ProteinSimple) supplemented with DMSO Inhibitor Mix (ProteinSimple) and Aqueous Inhibitor Mix (ProteinSimple) at 4 °C for 30 min, and the lysate was mixed with Premix G2 (pH 3–10) (ProteinSimple) and pI Standard Ladder 3 (44:1, ProteinSimple). The rabbit anti-NF-κB (p65) antibody (primary antibody, Cell Signaling Technology) was diluted 1:50 in antibody dilution buffer, and the anti-human IgG-HRP (secondary antibody, Protein Simple) was diluted 1:100 in antibody dilution buffer. Luminol/peroxide was mixed at a 1:1 ratio. The NanoPro 1000 (ProteinSimple) was loaded and run according to the manufacturer’s specifications. Emitted light was quantified for 30 s, 60 s, 120 s, 240 s, 480 s, and 960 s. Compass software 2.5.11 (ProteinSimple) was used to identify and quantify chemiluminescence peaks and optimize tracings.

### TNF-α-stimulated MSCs and LPS-stimulated MSCs

CD106^+^ MSCs or CD106^–^ MSCs at passage 4 were stimulated by incubation with TNF-α (PepTech, Burlington, MA, USA) 10 ng/ml for 1 h, 4 h, or 24 h, or 500 ng/ml LPS (Sigma-Aldrich) for 0.5 h, 1 h, 2 h, or 4 h.

### Statistical analysis

Analysis of variance in conjunction with Student’s *t* test was performed to identify significant differences. All analyses were performed using GraphPad Prism 6.0 software (GraphPad, La Jolla, CA, USA).

## Results

### Isolation and identification of BM-MSCs

BM-MSCs were isolated and cultured from AA patients and healthy controls and harvested at passage 4 to analyze immunophenotypes using FCM. Higher expression of CD13, CD29, CD44, CD49e, CD73 (SH3), CD90, CD105 (SH2), CD166, and human leukocyte antigen ABC (HLA-ABC), but not CD31, CD34, CD45, CD11b, CD14, CD40, and HLA-DR were expressed on the surface of BM-MSCs with no significant differences between AA patients and healthy controls (Additional file 1: Figure S1).

### Gene expression profile of BM-MSCs

To understand the molecular mechanisms underlying the deficiency of BM-MSCs in AA, we compared the gene expression profiles of MSCs from AA patients and healthy controls. We found that 1678 genes were differentially expressed in BM-MSCs from AA patients. Overall, 768 genes belonging to different functional categories and signaling pathways were upregulated and 910 genes were downregulated. We found that some of the differentially expressed genes are associated with the hematopoietic cell lineage, including osteoblastic, adipogenic, and endothelial differentiation. Among these genes, the expression of CD106 gene (VCAM1) was significantly different between AA patients and healthy controls (Additional file 4).

### Expression of CD106 on BM-MSCs from AA patients

**Fig. 1:**
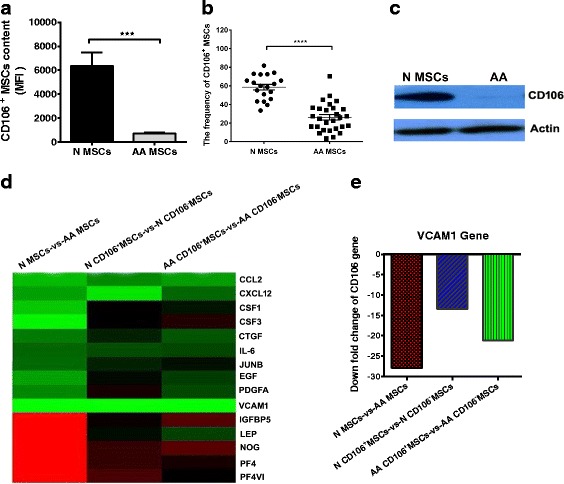
The expression of the CD106 gene and protein in mesenchymal stem cells (*MSCs*). Comparison of cluster data between aplastic anemia (*AA*) patients and healthy controls (*N*). **a** Significant reduction of the mean fluorescence intensity (*MFI*) of CD106^+^ MSCs observed in AA patients compared to controls. **b** Decreased frequency of CD106^+^ MSCs shown in AA patients (*n* = 28) in comparison with that of healthy controls (*n* = 19). **c** The protein expression of CD106 on MSCs by Western blot from AA patients and healthy controls. **d** Differential expression of 15 genes between AA patients and healthy controls, CD106^+^ MSCs and CD106^–^ MSCs from healthy controls, and CD106^+^ MSCs and CD106^–^ MSCs from AA patients. **e** Downfold change of the CD106 gene between AA patients and healthy controls, CD106^+^ MSCs and CD106^–^ MSCs from healthy controls, and CD106^+^ MSCs and CD106^–^ MSCs from AA patients. ***P < 0.001, **** *P*<0.0001. *CCL2* chemokine ligand 2, *CXCL12* C-X-C motif chemokine 12, *CSF* colony-stimulating factor, *CTGF* connective tissue growth factor, *IL* interleukin, *JUNB* JunB proto-oncogene, *EGF* epidermal growth factor, *PDGFA* platelet-derived growth factor alpha, *IGFBP5* insulin-like growth factor-binding protein 5, *LEP* leptin, *NOG* Noggin, *PF4* platelet factor 4, *PF4V1* platelet factor 4 variant 1

AA patients had significantly decreased mean fluorescence intensity (MFI) of CD106^+^ MSCs (713.7 ± 95.13 vs. 6351 ± 1125, *P* < 0.001) compared with healthy controls (Fig. 1a), and AA patients had significantly decreased frequencies of CD106^+^ MSCs (26.2 ± 2.9% vs. 58.7 ± 3.1%, *P* < 0.0001) compared with healthy controls (Fig. 1b).Western blotting revealed that the total CD106 protein expression in BM-MSCs from AA patients was significantly reduced compared with that of healthy controls (Fig. 1c).

### Gene expression profile of BM-CD106^+^ MSCs and BM-CD106^–^ MSCs

We then compared the gene expression in CD106^+^ MSCs and CD106^–^ MSCs. In comparison with CD106^+^ MSCs, 37 genes were differentially expressed (13 upregulated and 24 downregulated) in CD106^–^ MSCs from healthy controls, and 49 genes were differentially expressed (18 upregulated and 31 downregulated) in CD106^–^ MSCs from AA patients. We studied 15 genes–CCL2, CXCL12, colony-stimulating factor 1 (CSF1), CSF3, connective tissue growth factor (CTGF), IL-6, Jun B proto-oncogene (JUNB), epidermal growth factor (EGF), platelet-derived growth factor alpha (PDGFA), CD106 (VCAM1), insulin-like growth factor-binding protein 5 (IGFBP5), leptin (LEP), Noggin (NOG), platelet factor 4 (PF4), and PF4 variant 1 (PF4V1) associated with the hematopoietic cell lineage, including osteoblastic, adipogenic and endothelial differentiation—and found that expression of these genes was significantly different between BM-MSCs from AA patients and healthy controls as well as between CD106^+^ BM-MSCs and CD106^–^ BM-MSCs from both AA and healthy controls (Fig. 1d). The down fold change of CD106 gene (VCAM1) was shown (Fig. 1e). In particular, CD106 gene (VCAM1), CXCL12, CCL2, and IL-6 genes were found to be upregulated in unsorted BM-MSCs from healthy controls and in CD106^+^ MSCs from both AA patients and healthy controls. The expression of the selected genes determined using GeneChip detection was consistent with qRT-PCR results (Additional file 2: Figure S2).

### Angiogenic capacities of CD106^–^ MSCs in vitro and in vivo

**Fig. 2:**
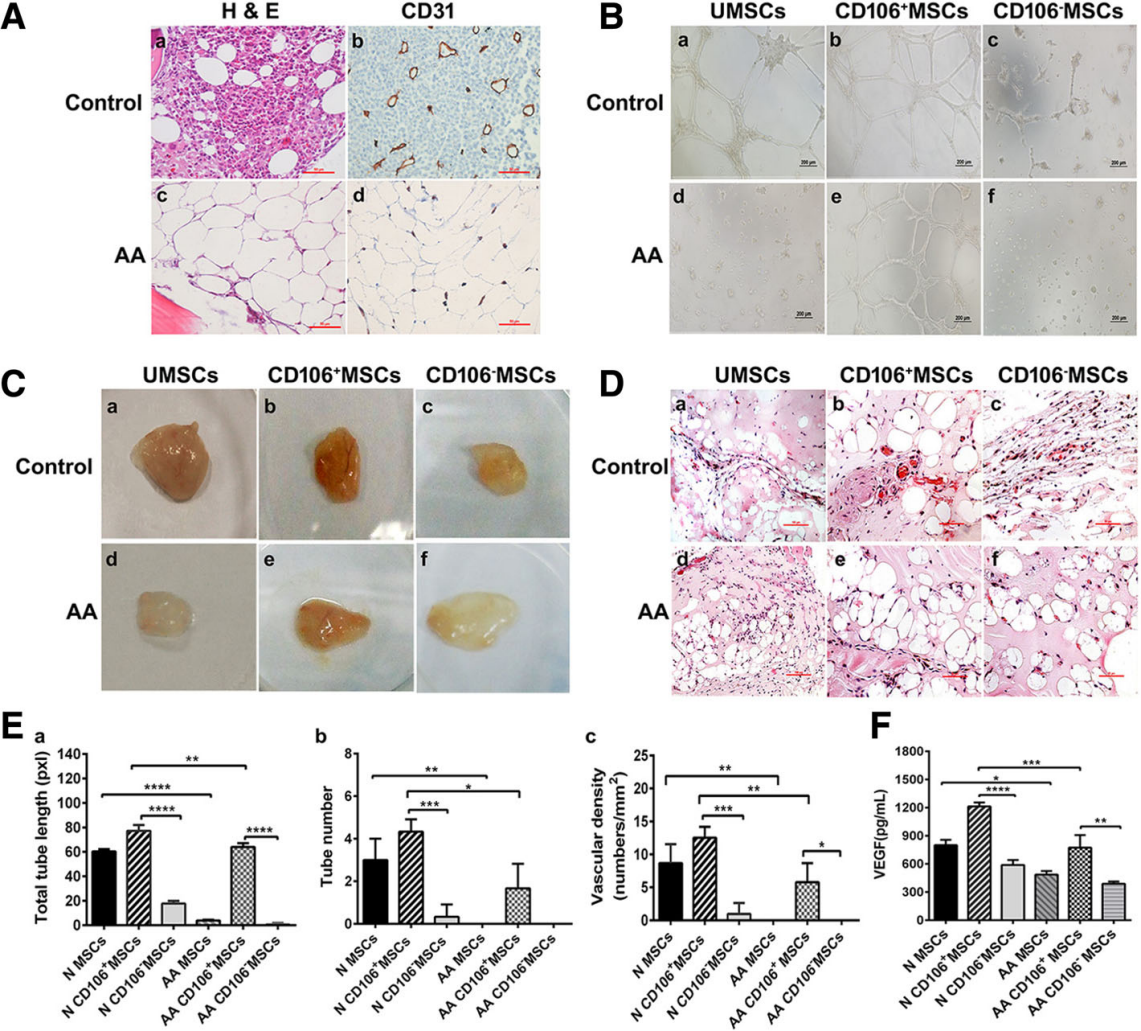
Impaired vasculogenesis ability of CD106^+^/CD106^–^ BM-MSCs from aplastic anemia (*AA*) patients and controls (*N*) in vivo and in vitro. **A** Hematoxylin and eosin (*H&E*) staining and histochemical staining (CD31) were applied to BM biopsies in AA patients and healthy controls, and the results showed that hematopoietic tissues and blood vessels (*brown tubular structure*) were significantly reduced in AA patients (c,d) than in controls (a,b). **B** Capillary tube-like formation of unsorted mesenchymal stem cells (*UMSCs*), CD106^+^ MSCs, and CD106^–^ MSCs from healthy controls (a, b, and c, respectively) and AA patients (d, e, and f, respectively). **C** In vivo vasculogenesis of unsorted MSCs, CD106^+^ MSCs, and CD106^–^ MSCs from healthy controls (a, b, and c, respectively) and AA patients (d, e, and f, respectively) using Matrigel plug assay. **D** In vivo vasculogenesis of unsorted MSCs, CD106^+^ MSCs, and CD106^–^ MSCs from healthy controls (a, b, and c, respectively) and AA patients (d, e, and f, respectively) using H&E staining. **E** (a) Quantification of capillary tube-like formation; (b) quantification of vasculogenesis using Matrigel plug assay; (c) quantification of in vivo vasculogenesis using H&E staining. **F** Levels of vascular endothelial growth factor (*VEGF*) in the supernatant of unsorted MSCs, CD106^+^ MSCs, and CD106^–^ MSCs from AA patients and healthy controls. *P < 0.05, **P < 0.01, ***P < 0.001, ****P < 0.0001

H&E (Sigma-Aldrich) staining and histochemical staining (CD31) were applied to BM biopsies from AA patients and healthy controls, and the results showed that hematopoietic tissues and blood vessels were significantly reduced in AA patients (Fig. 2A). The angiogenic capacity and hematopoietic-supporting activities of different populations of BM-MSCs were subsequently analyzed. The angiogenic capacity of unsorted MSCs, CD106^+^ MSCs, and CD106^–^ MSCs from AA patients and healthy controls was assessed using an in vitro Matrigel plug assay. BM-MSCs from AA patients had significantly reduced vasculogenesis capacity compared with those from healthy controls (Fig. 2B and Ea). CD106^+^ MSCs from both AA patients and healthy controls had greater tube length and tube area than CD106^–^ MSCs.

We further examined vasculogenesis capacity using an in vivo Matrigel plug assay. MSCs from healthy controls demonstrated higher microvessel densities than MSCs from AA patients (Fig. 2Ca and Cd and Eb).These results were confirmed by H&E staining (Fig. 2Da, Dd and Ec). CD106^+^ MSCs from both AA patients and healthy controls showed higher microvessel densities than CD106^–^ MSCs (Fig. 2C and Eb), which was confirmed by H&E staining (Fig. 2D and Ec).These results were in line with those obtained in vitro and demonstrated the lower vasculogenesis capacity of CD106^–^ MSCs.

The supernatant of CD106^+^ MSCs from both AA patients and healthy controls had higher levels of VEGF than that of CD106^–^ MSCs (773.69 ± 133.04 vs. 388.06 ± 23.03 for AA patients, 1212.97 ± 40.97 vs. 587.52 ± 53.84 for healthy controls) (Fig. 2F).

### CD106 deficiency impaired the hematopoietic-supporting activities of BM-MSCs from AA patients in vitro

**Fig. 3:**
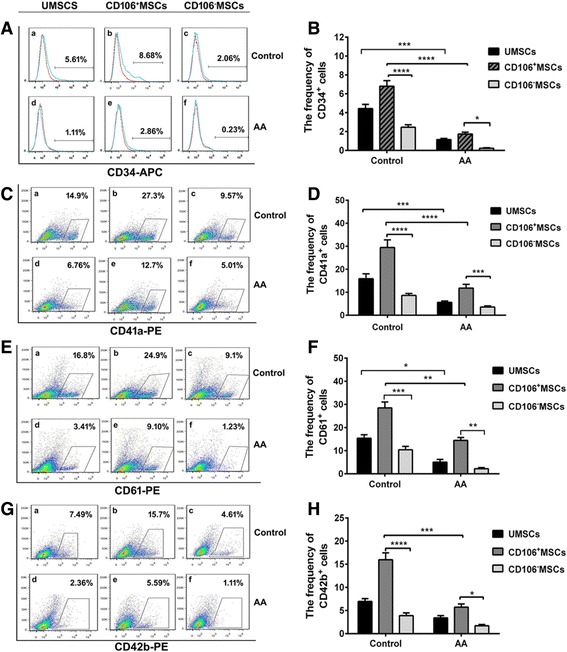
The capacity of CD106^+^/CD106^–^ BM-MSCs from AA patients and controls to support and maintain hematopoiesis in vitro. Proportion of CD34^+^ (**A** and **B**), CD41a^+^ (**C** and **D**), CD61^+^ (**E** and **F**), and CD42b^+^ (**G** and **H**) cells cocultured with unsorted mesenchymal stem cells (*UMSCs*) (a), CD106^+^ MSCs (b), and CD106^–^ MSCs (c) from healthy controls (*n* = 5) and unsorted MSCs (d), CD106^+^ MSCs (e), and CD106^–^ MSCs (f) from aplastic anemia (*AA*) patients (*n* = 5). Data are represented as the mean ± standard error. *P < 0.05, **P < 0.01, ***P < 0.001, ****P < 0.0001

To assess the capacity of BM-MSCs to support and maintain hematopoiesis, we cocultured sorted CD34^+^ cells with unsorted MSCs, CD106^+^ BM-MSCs, or CD106^–^ MSCs from AA patients or healthy controls to mimic the interactions between HPCs and the BM microenvironment in vitro. The percentage of CD34^+^ cells cocultured with unsorted BM-MSCs from AA patients was significantly lower than that of CD34^+^ cells cocultured with the same cells from healthy controls (*P* < 0.001) (Fig. 3Aa, Ad, and B), whereas the percentage of CD34^+^ cells cocultured with CD106^–^ MSCs was significantly lower than that of CD34^+^ cells cocultured with CD106^+^ MSCs from both AA patients (*P* < 0.05) and healthy controls (*P* < 0.0001) (Fig. 3A and B). Similar differences were observed in the percentage of CD41a^+^ cells (*P* < 0.001, *P* < 0.001, and *P* < 0.0001, respectively) (Fig. 3C and D), CD61^+^ cells (*P* < 0.05, *P* < 0.01, and *P* < 0.001, respectively) (Fig. 3E and F), and CD42b^+^ cells (*P* < 0.01, *P* < 0.05, and *P* < 0.0001, respectively) (Fig. 3G and H) cocultured with unsorted MSCs, CD106^+^ BM-MSCs, or CD106^–^ MSCs from AA patients or healthy controls.

**Fig. 4:**
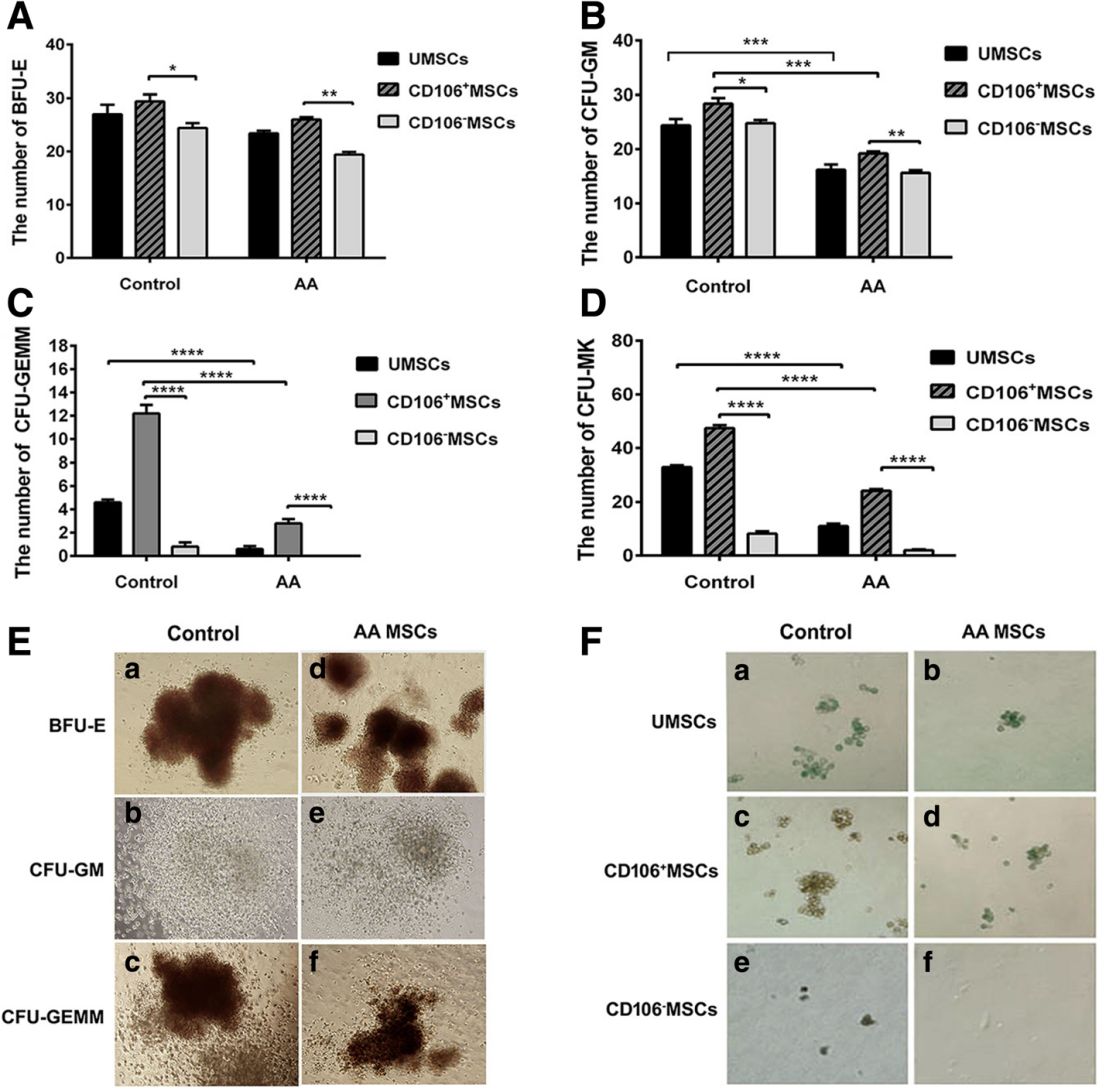
Impaired capacity of CD106^+^/CD106^–^ BM-MSCs from aplastic anemia (*AA*) patients and controls to support colony formation of UCB CD34^+^ cells. Purified CD34^+^ cell-derived colony formation of burst-forming unit-erythroid (*BFU-E*) (**A**), colony-forming unit-granulocyte macrophage (*CFU-GM*) (**B**), CFU mixed cell (*CFU-GEMM*) (**C**), and CFU-megakaryocyte (*CFU-MK*) (**D**) on a layer of unsorted mesenchymal stem cells (*UMSCs*), CD106^+^ MSCs, or CD106^–^ MSCs from healthy controls (*n* = 5) and AA patients (*n* = 5). **E** The shape of CFUs. The CFU-GM, BFU-E, and CFU-GEMM colonies in the presence of unsorted MSCs, CD106^+^ MSCs, or CD106^–^ MSCs from healthy controls (a, b, and c) and from AA patients (d, e, and f). **F** Impaired capacity of BM-MSCs from AA patients to promote the CFU-MK formation of UCB CD34^+^ cells. The size of the CFU-MK colony in the presence of unsorted MSCs, CD106^+^ MSCs, or CD106^–^ MSCs from healthy controls (a, c, and e) was significantly larger than that of CFU-MK in the presence of the corresponding MSCs from AA patients (b, d, and f). Data are represented as the mean ± standard error. *P < 0.05, **P < 0.01, ***P < 0.001, ****P < 0.0001

We then evaluated the proliferative potential of recovered CD34^+^ cells after 14 days of culture, which was estimated by their capacity to generate hematopoietic colonies in semisolid methylcellulose. Significantly lower numbers of burst-forming unit-erythroid (BFU-E), colony-forming unit-granulocyte macrophage (CFU-GM), and CFU-mixed cell (CFU-GEMM) colonies were observed when purified CD34^+^ cells were cultured for 14 days on a layer of unsorted BM-MSCs from AA patients (Fig. 4A, B, and C). The results showed that CD106^+^ MSCs had an increased capacity to support the growth of hematopoiesis compared with CD106^–^ MSCs from both AA patients and healthy controls (Fig. 4A, B, and C). We also observed BFU-E, CFU-GM, and CFU-GEMM colonies in the presence of BM-MSCs from AA patients (Fig. 4Ed, Ee, and Ef) and from healthy controls (Fig. 4Ea, Eb, and Ec).

A CFU-MK-specific assay revealed that CD106^+^ MSCs more strongly supported the formation of an MK colony of CD34^+^ cells than unsorted BM-MSCs and CD106^–^ MSCs from AA patients or healthy controls. CFU-MK colonies from AA patients were significantly decreased compared with those from healthy controls (*P* < 0.0001) (Fig. 4D). In addition, the size of CFU-MK colonies in the presence of unsorted BM-MSCs, CD106^+^ MSCs, and CD106^–^ MSCs from AA patients (Fig. 4Fb, Fd, and Ff) was significantly smaller than that of the same colonies in the presence of unsorted BM-MSCs, CD106^+^ MSCs, and CD106^–^ MSCs from healthy controls (Fig. 4Fa, Fc, and Fe). Examination with an inverted microscope confirmed the presence of CFU-MK colonies. These results indicated the important role of CD106^+^ MSCs in the growth of hematopoietic stem cells, and suggested that BM-MSCs from AA patients were deficient in maintaining hematopoiesis.

**Fig. 5:**
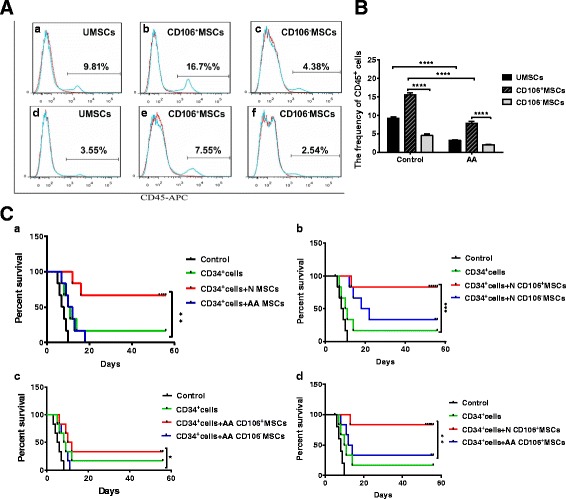
Impaired capacity of CD106^+^/CD106^–^ BM-MSCs from aplastic anemia (*AA*) patients and controls to support and maintain hematopoiesis and angiogenesis in xenotransplanted NOD/SCID mice. **A**,**B** The percentage of human CD45^+^ cells in mice transplanted with unsorted mesenchymal stem cells (*UMSCs*), CD106^+^ MSCs, or CD106^–^ MSCs from healthy controls (*n* = 5) (Aa, Ab, Ac, and B) and AA patients (*n* = 5) (Ad, Ae, Af, and B). **C** The survival of mice cotransplanted with UCB CD34^+^ cells and unsorted MSCs from healthy controls was superior compared with that of mice cotransplanted with same cells from AA patients (a). The survival of mice cotransplanted with UCB CD34^+^ cells and CD106^+^ MSCs was superior compared with that of mice cotransplanted with CD106^–^ MSCs from healthy controls (b) or AA patients (c). The survival of mice cotransplanted with UCB CD34^+^ cells and CD106^+^ MSCs from healthy controls was superior compared with that of mice cotransplanted with the same cells from AA patients (d). Data are represented as the mean ± standard error. **P < 0.01, ***P < 0.001, ****P < 0.0001

The UCB CD34^+^ cell engraftment in mice was examined 42 days after transplantation. The percentage of human CD45^+^ cells was significantly lower in recipient mice transplanted with unsorted BM-MSCs from AA patients than in mice transplanted with the same cells from healthy controls (*P* < 0.0001) (Fig. 5Aa, Ad and B). Similarly, the percentage of human CD45^+^ cells was significantly lower in recipient mice injected with CD106^–^ MSCs than mice with CD106^+^ MSCs from either AA patients (*P* < 0.0001) or healthy controls (*P* < 0.0001) (Fig. 5A and B). The survival of six additional recipient mice was observed until 56 days after xenotransplantation or death. The survival of mice cotransplanted with UCB CD34^+^ cells and unsorted BM-MSCs from healthy controls was superior to that of mice transplanted with BM-MSCs from AA patients (Fig. 5Ca). We then performed a comparative study to determine the subpopulation of BM-MSCs that better supports the survival of mice. Cotransplantation of UCB CD34^+^ cells and CD106^+^ MSCs or CD106^–^ MSCs from healthy controls prolonged the survival of mice compared with a single transplantation of UCB CD34^+^ cells (Fig. 5Cb). When mice were coinjected with UCB CD34^+^ cells and CD106^+^ MSCs or CD106^–^ MSCs from AA patients, a prolonged survival time was only observed in mice coinjected with UCB CD34^+^ cells and CD106^+^ MSCs (Fig. 5Cc). The best survival time was found in mice cotransplanted with UCB CD34^+^ cells and CD106^+^ MSCs from healthy controls (Fig. 5Cd).

### Expression and function of NF-κB (p65)

**Fig. 6:**
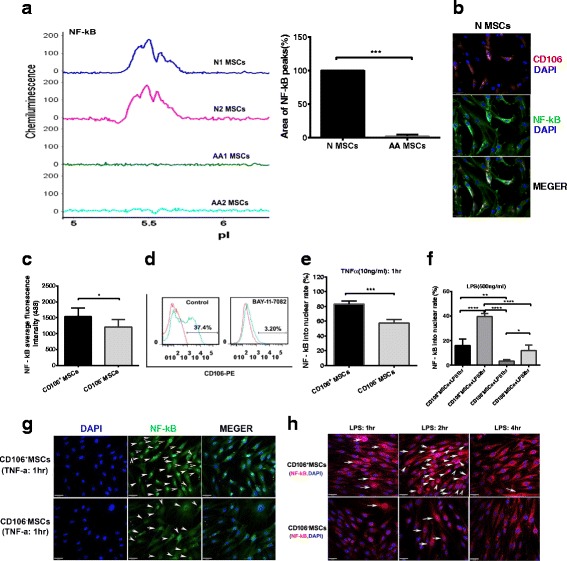
Expression and function of NF-κB (p65). **a** *Left panel*: NanoPro NF-κB profiles in BM mesenchymal stem cells (*MSCs*) from healthy controls (*N*) and aplastic anemia (*AA*) patients. *Right panel*: Quantitation of NF-κB in BM-MSCs from AA patients and healthy controls (*n* = 4). **b** Immunofluorescence on CD106 (*red*), NF-κB (*green*), and nuclei (*blue*) in unsorted MSCs from healthy controls. **c** The expression of NF-κB fluorescence was found in CD106^+^ BM-MSCs and CD106^–^ BM-MSCs from healthy controls. **d** The expression of CD106 was not found in BM-MSCs from healthy controls when blocked by a NF-κB specific inhibitor. **e** The ratio of NF-κB by tumor necrosis factor alpha (*TNF-α*) nuclear transfer stimulated at 1 h. **g** Immunofluorescence on NF-κB (*green*) and nuclei (*blue*) in CD106^+^ BM-MSCs and CD106^–^ BM-MSCs from healthy controls. **f** The ratio of NF-κB by lipopolysaccharide (*LPS*) nuclear transfer stimulated at 1 h and 2 h. **h** Immunofluorescence on NF-κB (*red*) and nuclei (*blue*) in CD106^+^ BM-MSCs and CD106^–^ BM-MSCs from healthy controls. *P<0.05 **P < 0.01, ***P < 0.001

A previous study has shown that NF-κB can selectively regulate CD106 (VCAM-1) gene expression in vivo [11]. Therefore, we detected the expression of NF-κB in BM-MSCs from AA patients and healthy controls and found that the profile (Fig. 6a, left) and quantitation (*P* < 0.001) (Fig. 6a, right) of NF-κB was decreased in BM-MSCs from AA patients compared with those in BM-MSCs from healthy controls. We also detected NF-κB in BM-MSCs from healthy controls, and found that CD106^+^ MSCs had a higher expression of NF-κB (Fig. 6b).

A higher expression of NF-κB was found in CD106^+^ BM-MSCs than in CD106^–^ BM-MSCs from healthy controls (1543 ± 88.82 vs. 1210 ± 106.7) (Fig. 6c; Additional file 3: Figure S3). CD106 expression was not found in BM-MSCs from healthy controls blocked by a NF-κB-specific inhibitor (Fig. 6d).

We then detected the ratio of NF-κB after TNF-α-induced nuclear transfer that was initiated at 1 h, 4 h, and 24 h. The ratio of NF-κB in CD106^+^ MSCs was higher than that in CD106^–^ MSCs (83.00 ± 2.08% vs. 57.25 ± 2.50%) from healthy controls at 1 h (Fig. 6e and g). No nuclear transfer phenomenon for either NF-κB or TNF-α stimulation was observed at 4 h and 24 h (Fig. 6e and g). We also detected the ratio of NF-κB after LPS-induced nuclear transfer that was initiated at 0.5 h, 1 h, 2 h, and 4 h. The ratio of NF-κB in CD106^+^ MSCs was higher than that in CD106^–^ MSCs (16.25 ± 2.53% vs. 3.30 ± 0.76%, and 39.75 ± 1.25 vs. 11.93 ± 2.21%) from healthy controls at 1 h and 2 h (Fig. 6f and h). No nuclear transfer phenomenon for NF-κB was observed at 0.5 h and 4 h (Fig. 6f and h).

### CD34^+^ cell maintenance capacity of BM-CD106^+^ MSCs with blocked NF-κB

**Fig. 7:**
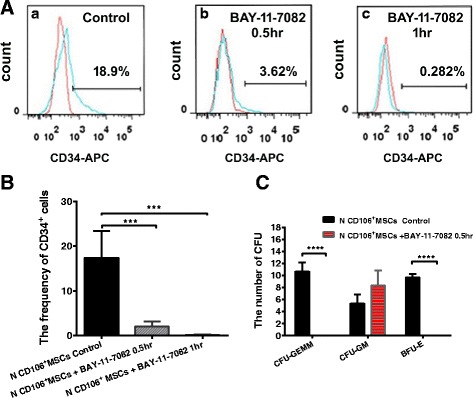
Effects of blocked CD106^+^ BM-MSCs by a NF-κB-specific inhibitor on cord blood CD34^+^ cells. **A**,**B** CD106^+^ BM-MSCs from healthy controls (*N*) blocked by the NF-κB-specific inhibitor and then cocultured with sorted CD34^+^ cells. The percentage of CD34^+^ cells in the controls (a) and in the groups of blocked NF-κB at 0.5 h (b) and 1 h (c). **C** The number of colony-forming unit-granulocyte macrophage (*CFU-GM*), burst-forming unit-erythroid (*BFU-E*), and CFU mixed cell (*CFU-GEMM*) colonies was observed when purified CD34^+^ cells were cultured for 14 days in the controls and in the groups of blocked NF-κB at 0.5 h. ***P < 0.001 ****P<0.0001

To determine the relationship between NF-κB and the CD106 (VCAM1) gene, we studied the role of CD106 (VCAM1) and NF-κB in supporting CD34^+^ cells. When CD106^+^ BM-MSCs from healthy controls were blocked by NF-κB-specific inhibitors and then cocultured with sorted CD34^+^ cells, the percentage of CD34^+^ cells was higher in the controls (17.40 ± 3.02%, *n* = 4) than in the groups with blocked NF-κB at 0.5 h (2.08 ± 0.54%, *n* = 4) and 1 h (0.16 ± 0.06, *n* = 4) (Fig. 7A and B).

We then evaluated the proliferative potential of recovered CD34^+^ cells after 14 days of culture by their capacity to generate hematopoietic colonies in semisolid methylcellulose. Only the CFU-GM colonies, but not the CFU-GEMM or BFU-E colonies, were observed when purified CD34^+^ cells were cultured for 10 days in a group in which CD106^+^ MSCs were blocked with BAY-11-7082 (0.5 h) (5.33 ± 0.88 vs. 8.33 ± 1.45 for CFU-GM) (Fig. 7C).

## Discussion

Acquired AA is characterized by deficiency or dysfunction of the BM microenvironment. However, little is known about the impairment of BM-MSCs in AA patients. Here, we reported that BM-MSCs from AA patients exhibited downregulation of CD106 in vitro and decreased potential for angiogenic and hematopoiesis-supporting abilities.

HSCs reside in complex, dynamic microenvironments or niches which are composed of supportive cells, extracellular growth factors, metabolic constituents, and matrix factors that actively regulate HSC function [10, 15–23] and enable a sustainable and responsive HSC pool [24]. Two physiologically distinct HSC niches have been identified in BM, the endosteal (or osteoblastic) niche at the BM interface and the vascular niche around specialized vascular endothelium [19]. Interactions between HSCs and niches are bidirectional. The niche regulates HSC self-renewal and fate-decisions, whereas HSCs modulate dynamic interactions between HSCs and their specialized BM microenvironments to coordinately preserve steady-state hematopoiesis and hematopoietic reconstitution [25].

Accumulating studies have shown that defective HSCs and a defective microenvironment may play important roles in AA [26–28]. BM angiogenesis was also found to be defective in acquired AA. Angioblasts can be derived from hemangioblasts and BM-MSCs [29, 30]. Previous studies reported that the microvessel density, serum VEGF levels, and VEGF expression are significantly lower in AA patients compared with healthy controls [31, 32]. All of these abnormalities were improved after successful immunosuppressive therapy or HSC transplantation [31, 32]. Moreover, the microvasculature has been described as a critical target in various disorders related to enhanced BM angiogenesis such as hematopoietic neoplasms [33, 34]. Thus, the vascular niche for HSCs in acquired AA might be defective in forming vessels and further decrease its hematopoietic-supporting activities. Further research is needed to clarify the vascular niche features in BM of acquired AA, which may provide valuable information for developing novel therapies.

Our previous study demonstrated that BM-MSCs from AA patients could easily be induced to differentiate into adipocytes but less easily into osteoblasts [13]. BM-MSCs from AA patients also exhibited impaired hematopoietic support. To understand the molecular mechanisms underlying the deficiency of BM-MSCs in AA, we compared the gene expression profiles of MSCs from AA patients and healthy controls and found a large number of differentially expressed genes; among them, the CD106 (VCAM1) gene was highly differentially expressed.

CD106 (VCAM1) serves as a ligand for very late antigen-4 (VLA-4), which is present on leukocytes [7, 35]. CD106 promotes strong adhesion of leukocytes to the endothelium [36]. Cell-to-cell adhesion mediated by CD106 is known to be critical for T-cell activation and leukocyte recruitment to inflammatory sites, and therefore it plays an important role in evoking effective immune responses. Our previous study showed that CD106 was highly expressed on chorionic villi (CV)-MSCs, moderately expressed on BM-MSCs, poorly expressed on umbilical cord MSCs, and not expressed on adipose MSCs. We also observed that TNF-α and IL-1β were required for expansion of CD106^+^ MSCs. There was a positive correlation between the expression of CD106 and the immunosuppressive effect of placental CV-MSCs, suggesting that CD106 could be used as a biomarker for a subpopulation of MSCs with unique immunosuppressive activity [8].

Based on these observations, we then sorted the BM-MSCs according to the surface molecular marker VCAM1 (CD106) and compared the gene expression profile of CD106^+^ MSCs and CD106^–^ MSCs from AA patients and healthy controls. The results showed that VCAM1 was upregulated in unsorted MSCs from healthy controls and in CD106^+^ MSCs from both AA patients and healthy controls. We further observed that the gene was associated with hematopoietic support and maintenance. The results revealed that the gene was downregulated in CD106^–^ MSCs. Deficiency of CD106^+^ MSCs might be responsible for the impairment of the BM microenvironment in AA.

To our knowledge, this is the first study showing a significant reduction in CD106^+^ MSCs in AA patients and we revealed that this reduction was associated with impaired function of BM-MSCs. CD106^–^ MSCs, but not CD106^+^ MSCs, from both AA patients and healthy controls displayed increased adipogenic differentiation capacity and reduced osteogenic differentiation capacity. CD106^–^ MSCs also showed impaired hematopoiesis, impaired capillary tube-like formation in vitro and vasculogenesis in vivo, and deficiency in hematopoietic-supporting activities both in vivo and in vitro.

CD106 (VCAM1) gene can be regulated by NF-κB, a pleiotropic regulator of gene expression [11]. Our results showed that the expression of NF-κB was decreased in BM-MSCs from AA patients in comparison with BM-MSCs from healthy controls. We also found that CD106^+^ MSCs had high expression of NF-κB. The expression of CD106 was not found in BM-MSCs blocked by the NF-κB-specific inhibitor BAY-11-7082. Higher expression of NF-κB was found in CD106^+^ MSCs than in CD106^–^ MSCs from healthy controls. We detected the ratio of NF-κB after TNF-α- and LPS-induced nuclear transfer and found higher activity of NF-κB in CD106^+^ MSCs than in CD106^–^ MSCs. When NF-κB was blocked, CD106^+^ MSCs exhibited impaired hematopoietic differentiation ability.

## Conclusions

Overall, our data demonstrate the role of bone marrow CD106^+^ MSCs and NF-κB functional deficiency in the pathogenesis of AA and suggest that novel therapeutic strategies in AA patients could be developed using CD106^+^ MSCs or NF-κB-based products.

## Additional files

Additional file 1: Figure S1. The expression of CD13, CD29, CD44, CD49e, CD73 (SH3), CD90, CD105 (SH2), CD166, and human leukocyte antigen ABC (HLA-ABC), but not CD31, CD34, CD45, CD11b, CD14, CD40, and HLA-DR, on the surface of BM-MSCs. (PDF 1193 kb)

Additional file 2: Figure S2. The expressions of the CD106 gene (VCAM1), CXCL12, CCL2, and IL-6 genes were detected by quantitative real-time polymerase chain reaction (qRT-PCR). (PDF 1180 kb)

Additional file 3: Figure S3. A higher expression of NF-κB was found in CD106^+^ BM-MSCs than in CD106^–^ BM-MSCs from healthy controls. (PDF 1145 kb)

Additional file 4: Gene data. The differently expressed genes detected by GeneChip. (XLSX 4 kb)

## Abbreviations

AA: Aplastic anemia
BFU-E: Burst-forming unit-erythroid
BM: Bone marrow
BSA: Bovine serum albumin
CCL: Chemokine ligand
CFU: Colony-forming unit
CFU-GEMM: Colony-forming unit mixed cell
CFU-GM: Colony-forming unit-granulocyte macrophage
CV: Chorionic villi
CXCL: C-X-C motif chemokine
ELISA: Enzyme-linked immunosorbent assay
FCM: Flow cytometry
H&E: Hematoxylin and eosin
HLA: Human leukocyte antigen
HPC: Hematopoietic progenitor cell
HRP: Horseradish peroxidase
HSC: Hematopoietic stem cell
IL: Interleukin
LPS: Lipopolysaccharide
MACS: Magnetic-activated cell sorting
MK: Megakaryocyte
MSC: Mesenchymal stem cell
N: Normal control
PBS: Phosphate-buffered saline
qRT-PCR: Quantitative real-time polymerase chain reaction
TNF: Tumor necrosis factor
UCB: Umbilical cord blood
VCAM1: Vascular cell adhesion molecule 1
VEGF: Vascular endothelial growth factor
