# Supplementary figures and images for: CD106 is a novel mediator of bone marrow mesenchymal stem cells via NF-κB in the bone marrow failure of acquired aplastic anemia
Source: Stem Cell Res Ther. 2017 Aug 1;8:178. doi: 10.1186/s13287-017-0620-4 (PMC5540520; doi:10.1186/s13287-017-0620-4)

# Figure S1

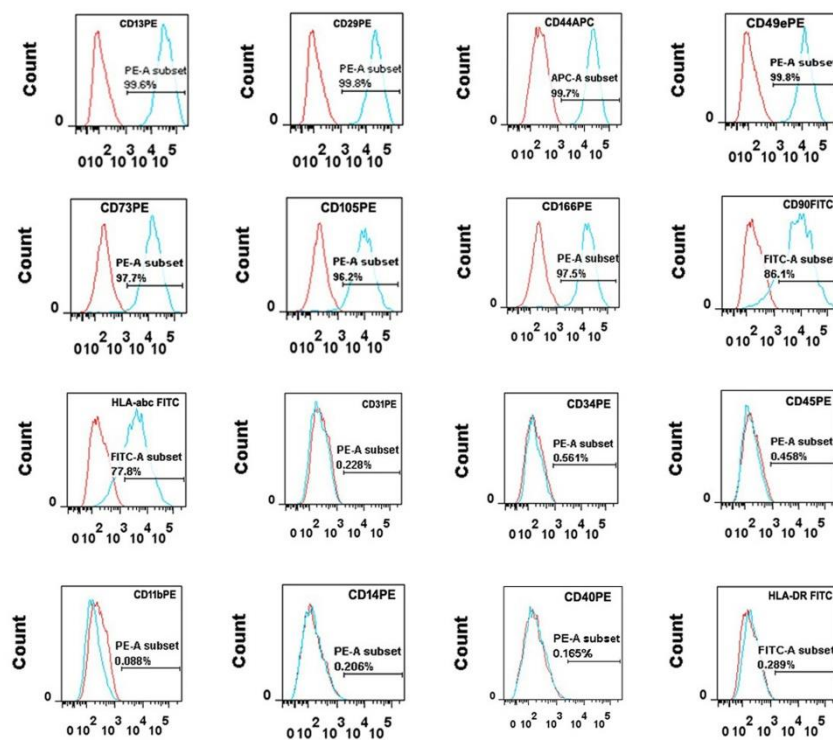

Supplement: Supplementary file 1 — The expression of CD13, CD29, CD44, CD49e, CD73 (SH3), CD90, CD105 (SH2), CD166, and human leukocyte antigen ABC (HLA-ABC), but not CD31, CD34, CD45, CD11b, CD14, CD40, and HLA-DR, on the surface of BM-MSCs. (PDF 1193 kb) [file 13287_2017_620_MOESM1_ESM.pdf]

**Figure S2**

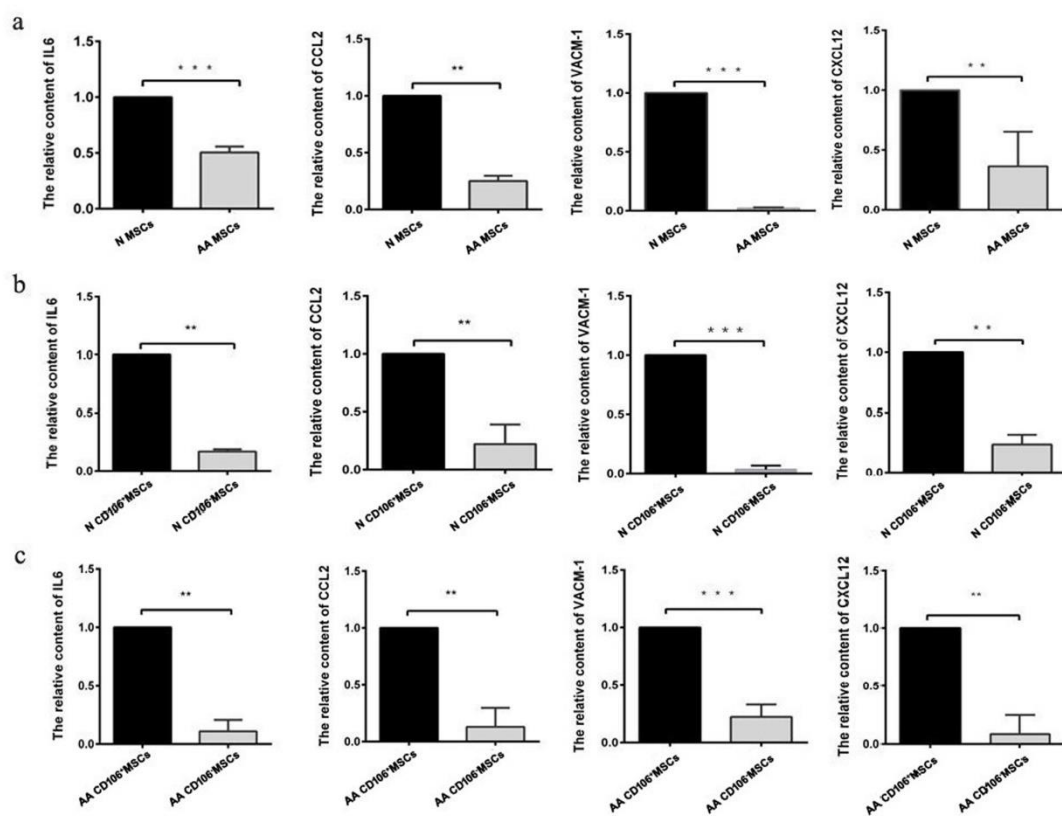

Supplement: Supplementary file 2 — The expressions of the CD106 gene (VCAM1), CXCL12, CCL2, and IL-6 genes were detected by quantitative real-time polymerase chain reaction (qRT-PCR). (PDF 1180 kb) [file 13287_2017_620_MOESM2_ESM.pdf]

# Figure S3

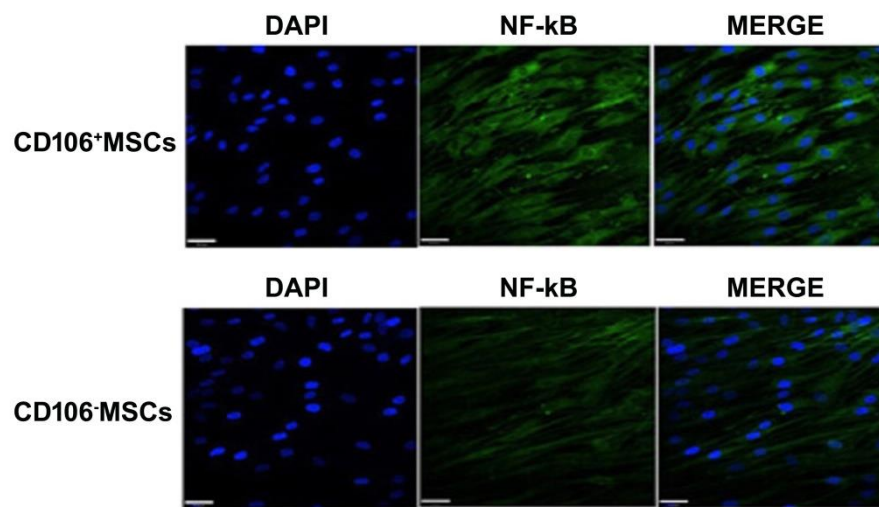

Supplement: Supplementary file 3 — A higher expression of NF-κB was found in CD106+ BM-MSCs than in CD106– BM-MSCs from healthy controls. (PDF 1145 kb) [file 13287_2017_620_MOESM3_ESM.pdf]
